# The effect of peroperative ultrasound used by the surgeon in parathyroidectomy on operation time

**DOI:** 10.1007/s00405-025-09319-7

**Published:** 2025-03-21

**Authors:** Mehmet Turan Cicek, Mehmet Aslan, Cigdem Firat Koca

**Affiliations:** Department of Otolaryngology Head and Neck Surgery, Faculty of Medicine, Malatya Inonu University, Malatya, Turkey

**Keywords:** Parathyroid adenoma, Minimally invasive parathyroidectomy, Ultrasonography

## Abstract

**Purpose:**

One of the endocrine conditions that endocrinologists see the most frequently is primary hyperparathyroidism, which is brought on by a parathyroid adenoma. Minimally invasive parathyroidectomy (MIP) has become the first line of treatment for primary hyperparathyroidism due to solitary parathyroid adenoma. Surgeon-performed ultrasonography (SUS), which has been found to be extremely accurate in localizing aberrant glands, has been employed preoperatively to augment the sensitivity of high-resolution ultrasonography (hUS).

**Methods:**

Two groups were randomly created from cases that underwent parathyroid surgery. In the first group, surgery was performed on 31 patients, taking into account the ultrasound results reported by radiology and the Technetium-99 m sestamibi scintigraphy results interpreted by the nuclear medicine clinic. In addition to the ultrasound results reported by radiology and the scintigraphy results interpreted by the nuclear medicine clinic, surgery was performed on 18 patients in the second group, that the surgeon applied ultrasound to them before the operation and separately evaluating the location of the parathyroid adenoma. Surgery time, hospital stay, pre and postoperative calcium and parathormon values were compared between the two groups.

**Results:**

When groups 1 and 2 were compared in terms of surgical time and hospital stay, there was a statistically significant difference between the two groups (*p* < 0.05). In Group 2, the duration of operation and hospital stay was significantly shortened. There was no statistically significant difference between the groups in terms of pre- and postoperative calcium and parathormone values (*p* > 0.05).

**Conclusions:**

We believe that the time required for surgery after a radiologist and surgeon performing ultrasonography is less than that required for surgery after radiologist performed ultrasonography demonstrating the efficacy of the surgery plus a radiologist and surgeon performing ultrasonography combination. We determined that the surgery time was statistically shorter in patients who underwent a radiologist and surgeon performing ultrasonography.

## Introduction

One of the endocrine conditions that endocrinologists see the most frequently is primary hyperparathyroidism, which is brought on by a parathyroid adenoma secreting excessive levels of parathyroid hormone (PTH) and consequently hypercalcaemia [1]. Primary hyperparathyroidism (pHPT) is thought to affect 1 in 500 women and 1 in 2,000 men, and its incidence is rising [2]. Primary hyperparathyroidism can result from either parathyroid hyperplasia or a parathyroid adenoma, however the underlying pathophysiology of the development of parathyroid adenomas is still unknown. Examples of these hereditary diseases include multiple endocrine neoplasia type 1 and type 2 [3]. Bilateral neck exploration of all four parathyroid glands is the standard surgical technique, which has a 95% success rate and very little morbidity [4–7]. However, 85–90% of individuals have a single adenoma as the etiology of pHPT [4, 8–9]. Parathyroid Technetium-99 m sestamibi scintigraphy (SS) and high-resolution ultrasonography (hUS) are two emerging technologies that make minimally invasive parathyroidectomy (MIP) the preferred procedure. The ability of the surgeon to carry out common surgical operations in creative ways in order to reduce the trauma of the surgical exposure is known as MIP [10, 11]. Potential benefits of MIP include shorter hospital stays, shorter operating times, less physical invasiveness, and improved cosmetic outcomes, given the safety and high success rate of bilateral neck exploration [10]. However, precise preoperative localization of parathyroid adenomas is necessary for the effectiveness of MIP [12–14]. Currently, the most popular preoperative localization modalities are hUS and SS. Between 70% and 90% of parathyroid adenomas can be identified with hUS and SS [6, 15, 16]. The accuracy of the anatomical localization of parathyroid adenomas is increased when 99mTc-sestamibi scintigraphy is combined with a single photon emission computerized tomography (SPECT) camera and merged with computed tomography (CT) (99mTc-sestamibi SPECT/CT). The usage of radioisotopes, slow scan times, poor anatomical localization when two glands are adjacent to one another, and the fact that many hospitals lack nuclear medicine departments are some possible disadvantages of this approach. This has led to the identification of parathyroid adenomas in B-mode pictures using high-frequency ultrasound scanning of the neck. Hypoechoic lesions located posteriorly and either superiorly or inferiorly to the thyroid gland’s poles are indicative of parathyroid adenomas. Among the many benefits of ultrasound are its ability to prevent ionizing radiation, its accessibility in all hospitals, and its precision in anatomical localization. The use of 99mTc-sestamibi SPECT/CT pre-operatively in all patients with peri-operative localization of the parathyroid gland utilizing high-frequency ultrasound using a portable ultrasound equipment is a solution to the problem mentioned above [3, 17]. Imaging study results will always be negative even though hUS and SS have a high sensitivity for single parathyroid adenomas [6]. Because of its affordability, ease of use, minimal radiation exposure, and capacity to assess concurrent thyroid nodules, hUS is preferred; yet, it might not be able to identify ectopic parathyroid adenomas [6, 16, 18]. Additionally, it has the drawback of being operator-dependent, with sensitivity levels among sonographers varying from 20 to 79% based on interest, experience, and skill [19–21]. SUS, which has been found to be extremely accurate in localizing aberrant glands, has been employed preoperatively to augment the sensitivity of hUS [22–26]. In this present study our aim was to evaluate the effect of peroperative SUS on operation time. The study included 49 patients undergoing parathyroid exploration.

## Materials and methods

49 patients who underwent parathyroidectomy surgery due to parathyroid adenoma (39 female, 10 male) in our clinic between 2018 and 2024 were included in the study, and historical patient records were analyzed. The study was conducted after obtaining approval from the ethics committee of the InonuUniversity Scientific Research and Publishing Ethics Board (Ethical approval number:2024/79 ). All patients received a preoperative laryngoscopic evaluation to assess vocal cord functions. Age, sex, the diagnosis, number of previous operations, operation type, preoperative imaging, complications, pre and postoperative calcium, parathormone values, ultrasonography result, Technetium-99 m sestamibi scintigraphy result, localization of adenoma( We classified the location of parathyroid adenomas as upper and lower left and right according to the thyroid site, and as ectopic outside the thyroid site) and operation times were all recorded. Parathyroid adenoma patients are referred to our clinic for surgery from the thyroid outpatient clinic of our hospital’s endocrinology department. All patients are referred to the thyroid clinic for routine blood tests, neck ultrasonography, Technetium-99 m sestamibi scintigraphy. Neck computerized tomography and/or neck magnetic resonance imaging are performed on patients whose location cannot be determined by USG and/or scintigraphy. Two groups were randomly created from cases that underwent parathyroid surgery. In the first group, surgery was performed on 31 patients, taking into account the ultrasound results reported by radiology and the Technetium-99 m sestamibi scintigraphy results interpreted by the nuclear medicine clinic. In addition to the ultrasound results reported by radiology and the scintigraphy results interpreted by the nuclear medicine clinic, surgery was performed on 18 patients in the second group, that the surgeon applied ultrasound to them before the operation and separately evaluating the location of the parathyroid adenoma. Surgery time and hospital stay were compared between the two groups. Patients under the age of 18, patients who had previously undergone thyroidectomy surgery, and patients who had additional thyroid surgery accompanying parathyroid adenoma were excluded from the study.

### Statistical analysis

The data obtained in the study were evaluated on a computer using the SPSS “Statistical Package For Social Sciences (SPSS22.0)” program. Ratio for qualitative variables, mean, standard deviation, median, minimum and maximum values ​​for quantitative variables were calculated. Pearson Chi-Square test was used for comparisons of qualitative variables. According to the Shapiro-Wilk test statistic (*p* > 0.05), parametric test statistics were used since the variables were suitable for normal distribution. According to the Independent Samples Shapiro-Wilk test statistic between the two groups (< 0.05), since the variables were not suitable for normal distribution, Mann Whitney U statistic, one of the non-parametric tests, was used. The results between the two groups were taken within the 95% confidence interval, and the statistical significance level was taken as *p* < 0.05.

## Results

**Fig. 1:**
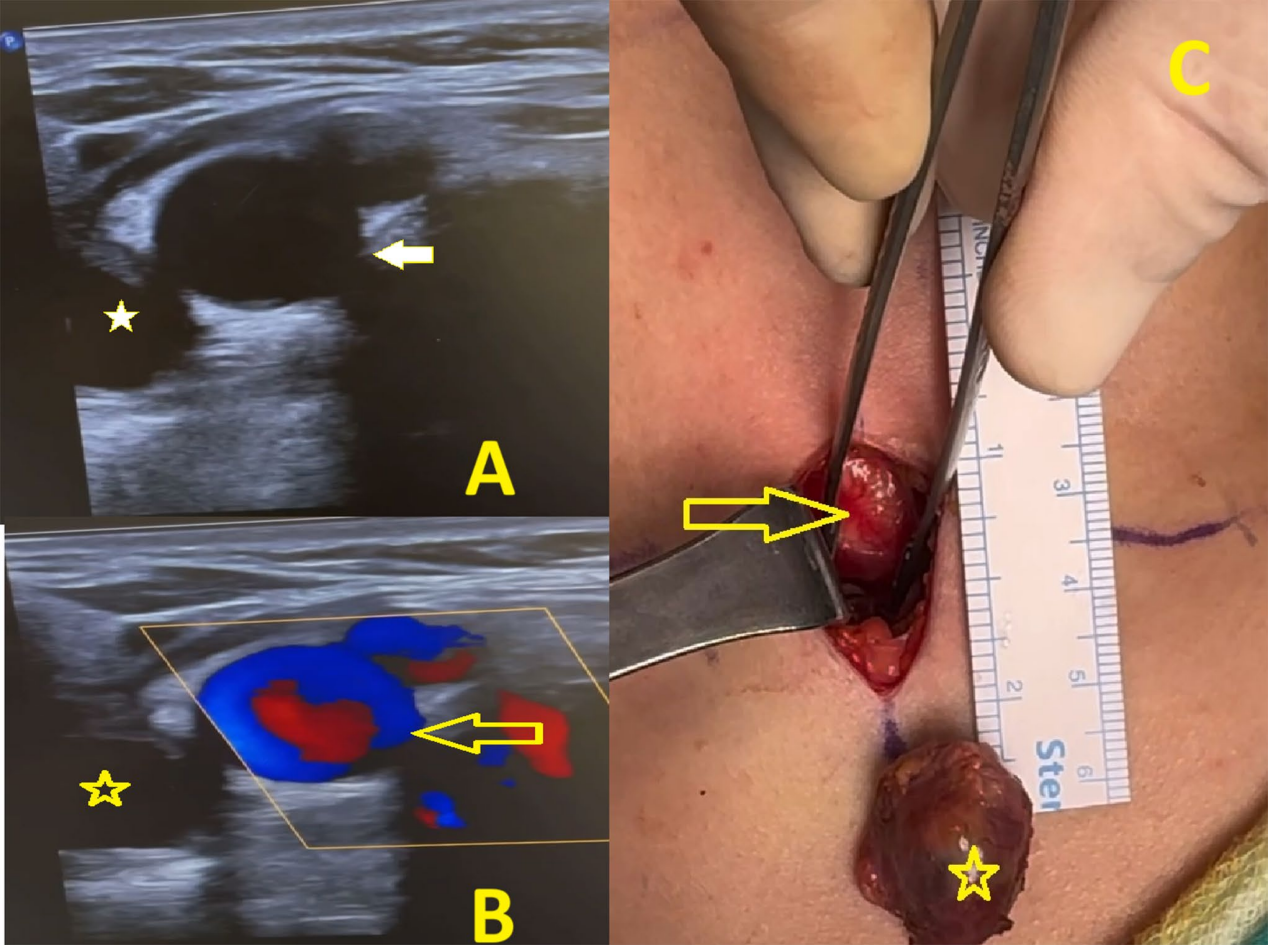
An ectopic parathyroid adenoma. **A**: Neck ultrasonography (surgeon-performed) sagittal section, white arrow right brachiocephalic- common carotid arteria * right inferior ectopic parathyroid adenoma. **B**: Neck doppler ultrasonography (surgeon-performed) sagittal section, yellow arrow right brachiocephalic common carotid arteria, * right inferior ectopic parathyroid adenoma. **C**: Intraoperative görüntü, yellow arrow brachiocephalic arteria, * parathyroid adenoma

Of the 49 patients we included in our study, 39 were female (79.5%) and 10 (20.5%) were male. The average age was calculated as 54.48 in Group 1 and 51.77 in Group 2. The most common location of parathyroid adenoma was in the lower left neck (54.8%) in Group 1 (Fig. 1) and in the lower right (44.4%) in Group 2. Ultrasonography performed by the radiologist correctly identified the location of the parathyroid adenoma in 27 of 31 patients (87.1%) in Group 1, and in 13 of 18 patients (72.2%) in Group 2. Technetium-99 m sestamibi scintigraphy method correctly identified adenoma in 16 of 31 patients (51.6%) in Group 1 and in 11 of 18 patients (61.1%) in Group 2.

**Fig. 2:**
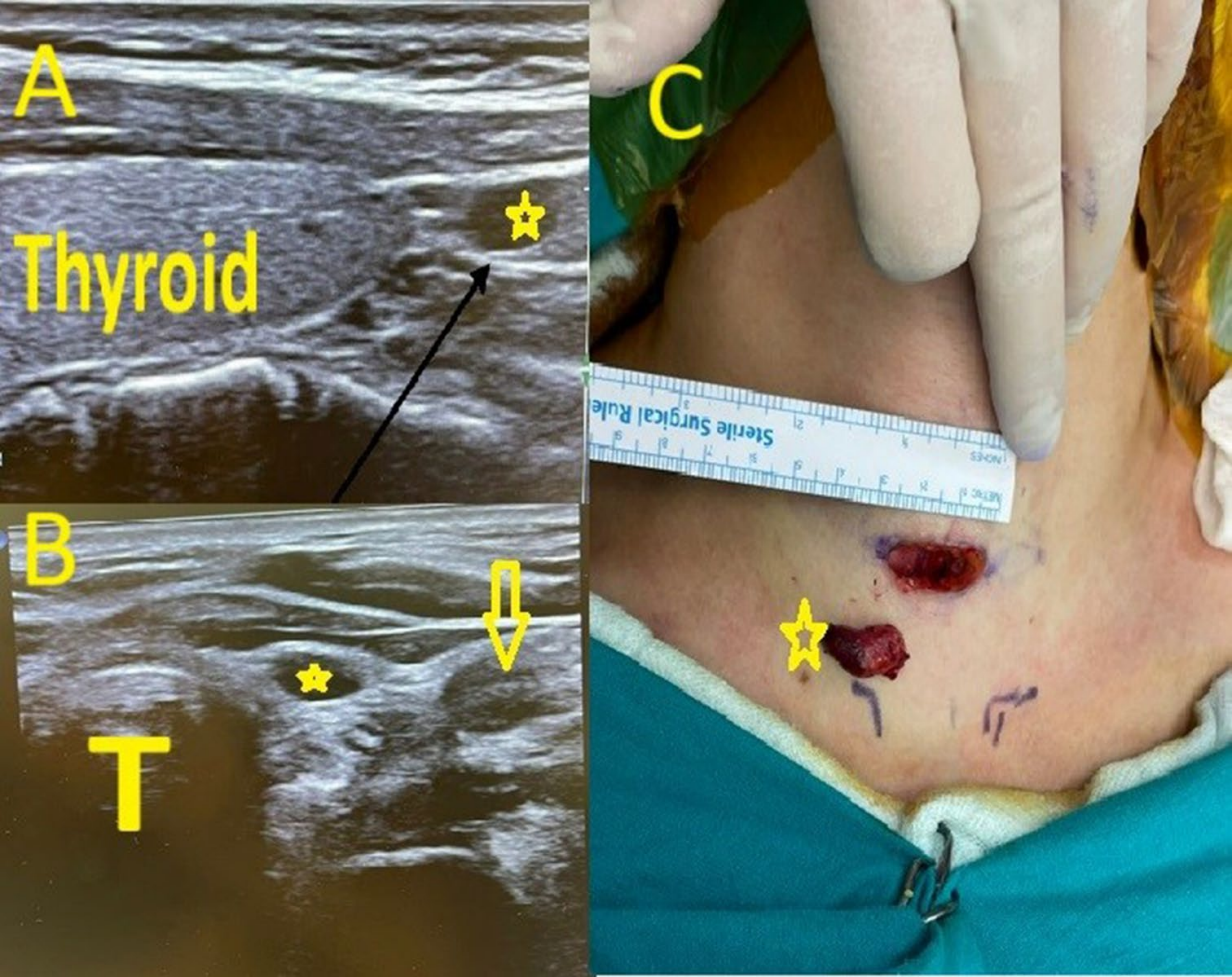
A left inferior parathyroid adenoma. **A**: Neck ultrasonography( surgeon-performed) sagittal section, * left inferior parathyroid adenoma. **B**: Neck ultrasonography axial section, * left inferior parathyroid adenoma, T trachea, yellow arrow left common carotid arteria. **C**: Intraoperative, * parathyroid adenoma

The adenoma was located ectopically in 1 patient in Group 2 and in 2 patients in Group 2. In 1 patient It was located at the carotid bifurcation (Fig. 2), in the tracheoesophageal groove in one patient, and retrosternally in 1 patient. In these 2 patients in the Group 2, the adenoma could not be localized by scintigraphy and ultrasonography, and the adenoma was detected by neck magnetic resonance imaging method. In these two patients, the location of the adenoma could not be localized in the ultrasonography performed by the surgeon. No statistically significant difference was found between group 1 and group 2 in terms of gender, side, accurate detection of adenoma in USG and scintigraphy (*p* > 0.05)( Table 1). When groups 1 and 2 were compared in terms of surgical time and hospital stay, there was a statistically significant difference between the two groups (*p* < 0.05). In Group 2, the duration of operation and hospital stay was significantly shortened. There was no statistically significant difference between the groups in terms of pre- and postoperative parathormone and postperative calcium values (*p* > 0.05)( Table 2).

**Table 1:** Demographics, adenoma localizations and radiologist-performed ultrasonography(rp Usg) and scintigraphy true identification rates

	Group 1		Group2			
	*n*	%	*n*	%	Test	*P* value
**Sex**					Fisher’s Exact Test	0,468
Female	26	83,9%	13	72,2%		
Male	5	16,1%	5	27,8%		
**Localization**					Fisher’s Exact Test 6,77	0,12
Right upper	3	9,7%	0	%		
Left upper	2	6,5%	3	16,7%		
Right lower	8	25,8%	8	44,4%		
Left lower	17	54,8%	5	27,8%		
Ectopic	1	3,2%	2	11,1%		
**rpUsg definition**					Fisher’s Exact Test	0,259
Yes	27	87,1%	13	72,2%		
No	4	12,9%	5	27,8%		
**Scintigraphy definition**					Pearson Chi-Square 0,415	0,519
Yes	16	51,6%	11	61,1%		
No	15	48,8%	7	38,9%		

Chi-Square Tests *p*<0,05

**Table 2:** Comparison of pre ve postoperative parathormon (Pth) values, postoperative calcium levels, hospital stay and surgery time

	Group	Group1	Group2	Test value	p
**Ca** ^++^	Mean	12,9032	9,9778	-0,354	0,72
	Median	10,1000	9,9000		
	Minimum	8,20	8,80		
	Maximum	101,00	11,70		
	Interquartile Range	1,10	,80		
**Pth preop**	Mean	355,8323	240,6500	-0,35	0,43
	Median	219,0000	171,0000		
	Minimum	47,80	48,00		
	Maximum	3131,00	840,00		
	Interquartile Range	261,00	186,83		
**Pth postop**	Mean	57,7935	41,2111	-0,778	0,19
	Median	55,0000	23,2000		
	Minimum	6,40	1,90		
	Maximum	190,00	109,00		
	Interquartile Range	61,00	75,68		
**Surgery time**	Mean	68,4516	43,0000	-4,628	**0,0001**
	Median	66,0000	40,0000		
	Minimum	48,00	26,00		
	Maximum	118,00	67,00		
	Interquartile Range	15,00	13,25		
**Hospital stay**	Mean	2,8065	2,3333	-2,257	**0,02**
	Median	3,0000	2,0000		
	Minimum	1,00	1,00		
	Maximum	4,00	7,00		
	Interquartile Range	1,00	2,00		

## Discussion

The return of calcium levels to normal following parathyroid surgery indicates the effectiveness of the procedure. However, it shouldn’t be viewed as the exclusive measure of success. Additionally, little morbidity, no mortality, low rates of recurrence, and a fair cost should all be associated with surgery [27].MIP lowers the rate of recurrent laryngeal nerve injury by preventing needless mobilization of neck tissues [28, 29]. In recent years, minimally invasive parathyroidectomies (MIP) have become increasingly common. Most people with primary hyperparathyroidism require surgery to remove the aberrant gland because they have increased serum levels of calcium and parathyroid hormone. To identify the aberrant gland, a highly precise imaging technique is required. For MIP, 99mTc-Sestamibi SPECT is not as anatomically accurate as it should be. Here, bedside ultrasonography in the clinic and the period right before the incision are helpful [30]. By extending the head and neck examination with ultrasound, the surgeon obtains a plethora of data that was previously limited to the radiology report’s text or a monitor in a radiology suite located distant from the operating room or surgeon’s office. It should come as no surprise that data obtained during a thyroid ultrasound by a surgeon can occasionally differ from data obtained by radiologists or ultrasound technicians [31]. When the operating surgeon uses ultrasonography, they can directly see the parathyroid adenoma, capture real-time images in several planes during the preoperative phase, and correlate the images with the thyroid and other neck landmarks [32]. We have shown that ultrasonography conducted by the surgeon during the immediate pre-operative phase offers extra detection of cases in which radiologist-performed ultrasound and 99mTc-sesta mibi failed to locate the lesion. Except for two ectopically located adenomas, the location of the adenoma was correctly determined by ultrasonography performed by the surgeon in all patients in group 2 (88.8%). Operative findings, postoperative calcium levels and hormone dynamics determined successful localization.The surgeon’s localization of parathyroid adenoma in our study is consistent with findings from other investigations. All of the patients in our series underwent first-line investigations that included sestamibi scintigraphy and/or ultrasound conducted by a radiologist prior to surgery. In our series, the sensitivity of sestamibi scintigraphy and ultrasound conducted by radiologists was 51.6% and 87.1%, for Group 1 and 61.1% and 72.2% for Group 2 respectively. Although our ultrasonography sensitivity was similar to the results of the meta-analysis conducted by CheungK, et al., our scintigraphy sensitivity was lower [33]. On the other hand, while our scintigraphy sensitivity result was similar to the study conducted by Aspinall et al., our ultrasonography sensitivity result performed by the radiologist was much higher [34]. Soon et al. examined 87 cases and found that 83% of patients were appropriately localized by peri-operative scan, whereas Untch et al. found that 87% of patients were correctly localized by scan in a clinic environment [19, 32]. Additional research has discovered comparable findings, such as those of van Ginhoven et al., who showed an accuracy of 85% [35]. Solorzano and associates showed that SUS was useful and a 67% accuracy rate in patients with non localizing MIBI scans [36]. Steward et al. showed that sensitivities for exact quadrant definition for ultrasound versus sestamibi were 87% versus 58% [37]. According to a study conducted in the UK by Aspinall et al., SUS in outpatient clinics showed an 86% accuracy rate in identifying the location of parathyroid adenomas [38]. When department ultrasonography is performed by a radiologist, the detection rates of adenomas vary significantly. Although the technique for ultrasound scans depends on the operator, surgeons seem to do better than radiologists, which may be due to factors like the surgeon’s experience in this field [3]. It is possible to find parathyroid adenomas in ectopic neck sites. The retroesophageal, paraesophageal, intra-thyroid, or thymus are common sites. Three ectopic adenomas were discovered in carotid bifurcation, tracheoesophageal sulcus and retrosternal sites during our investigation. Two of the adenomas could not be detected neither by SUS nor by ultrasonography performed by the radiologist and scintigraphy. While sestamibiscintigraphy is generally thought to be preferable for identifying ectopic adenomas, ultrasound has been demonstrated to be able to identify certain intra-thyroid ectopic glands [39, 40].An ectopic adenoma should be suspected if an ultrasonography scan is negative.

Ultrasound is a readily available, low-cost, non-invasive imaging technique. Ultrasonography training is becoming increasingly widespread and accessible, It is known that certain factors, like small gland size, obesity, multinodular thyroid goitres, and ectopic placement, can make it harder to detect adenomas on ultrasound [41]. Additionally, SUS ultrasonography enables the neck incision to be planned, which improves operation safety [42]. The duration of surgery and anesthesia is shortened by MIP [29, 43, 44]. The average MIP operating time reported in the literature is between 15 and 56 min [45, 46]. Additionally, it reduces hospital stays [28]. As a result, we believe that the time required for surgery after a radiologist and surgeon performing ultrasonography is less than that required for surgery after radiologist performed ultrasonography demonstrating the efficacy of the surgery plus a radiologist and surgeon performing ultrasonography combination. We determined that the surgery time was statistically shorter in patients who underwent a radiologist and surgeon performing ultrasonography. Mean surgery time was 43 min in this group.The duration of hospital stay was also statistically significantly shorter in Group 2. Uslukaya et al. stated this period as 1 day, patients were discharged after an average of two days in our trial [26].

This study has some limitations. First, neither radiologists nor surgeons who performed ultrasounds were blinded to the sintigraphy results. Second, a weakness of the study was the small number of patients that were enrolled.

If we consider our study from different aspects, the ultrasound in the surgeon’s hand was able to determine the location of the adenoma at a rate of 88.8%. In our study, we found adenoma intraoperatively in all patients and removed it without any problems. There was no postoperative problem. There was a decrease in parathyroid hormone levels in all of them. When evaluated as accurate surgery, our success rate can be stated as 100%.The ability to locate and identify aberrant glands is essential to the success of MIP. MIP results in a smaller incision, less dissection, less postoperative pain, and a shorter hospital stay. Because of the patient selection and successful treatment of the chosen patients, it is crucial that surgeons join ultrasonography. We believe that radiologists and surgeons complement one another. Furthermore, a radiologist and surgeon performing ultrasonography is the reason for our high success rate.
